# Supplementary material for: Single Cell Transcriptomic and Chromatin Profiles Suggest Layer Vb Is the Only Layer With Shared Excitatory Cell Types in the Medial and Lateral Entorhinal Cortex
Source: Front Neural Circuits. 2022 Jan 26;15:806154. doi: 10.3389/fncir.2021.806154 (PMC8826650; doi:10.3389/fncir.2021.806154)
Supplement: Supplementary file 14 [file Data_Sheet_1.ZIP › Exc_vs_Inh_Motifs/homerResults/motif11.similar.html]

motif11

## Information for motif11

C
G
T
A
A
C
G
T
G
T
A
C
A
G
T
C
A
T
G
C
G
T
C
A
A
G
T
C
C
T
G
A
  
Reverse Opposite:  

G
A
C
T
A
C
T
G
A
C
G
T
A
T
C
G
A
C
T
G
A
C
T
G
C
G
T
A
A
C
G
T
  

|  |  |
| --- | --- |
| p-value: | 1e-226 |
| log p-value: | -5.218e+02 |
| Information Content per bp: | 1.821 |
| Number of Target Sequences with motif | 21286.0 |
| Percentage of Target Sequences with motif | 50.46% |
| Number of Background Sequences with motif | 4638.5 |
| Percentage of Background Sequences with motif | 42.67% |
| Average Position of motif in Targets | 342.4 +/- 254.7bp |
| Average Position of motif in Background | 309.4 +/- 248.3bp |
| Strand Bias (log2 ratio + to - strand density) | 0.0 |
| Multiplicity (# of sites on avg that occur together) | 1.35 |
| Motif File: | file (matrix) reverse opposite |

### Similar de novo motifs found

|  |  |  |  |  |  |  |  |
| --- | --- | --- | --- | --- | --- | --- | --- |
| Rank | Match Score | Redundant Motif | P-value | log P-value | % of Targets | % of Background | Motif file |
| 1 | 0.655 | A C G T A C T G C G T A A C T G A C T G A C G T C G T A C G A T A T G C A G T C | 1e-60 | -139.026479 | 0.41% | 0.09% | motif file (matrix) |
